# ResolvinD1 Protects the Airway Barrier Against Injury Induced by Influenza A Virus Through the Nrf2 Pathway

**DOI:** 10.3389/fcimb.2020.616475

**Published:** 2021-02-12

**Authors:** Yan Guo, You-Hui Tu, Xu Wu, Shuang Ji, Ji-Long Shen, Hui-Mei Wu, Guang-He Fei

**Affiliations:** ^1^ Department of Respiratory and Critical Care Medicine, First Affiliated Hospital of Anhui Medical University, Hefei, China; ^2^ Key Laboratory of Respiratory Diseases Research and Medical Transformation of Anhui Province, First Affiliated Hospital of Anhui Medical University, Hefei, China; ^3^ Department of Pathogen Biology and Provincial Laboratories of Pathogen Biology and Zoonoses, Anhui Medical University, Hefei, China; ^4^ Anhui Geriatric Institute, Department of Geriatric Respiratory and Critical Care, First Affiliated Hospital of Anhui Medical University, Hefei, China

**Keywords:** resolvinD1, influenza A virus, primary bronchial epithelial, airway barrier, Nrf2, inflammation reaction

## Abstract

Airway barrier damage and excessive inflammation induced by influenza A virus (IAV) are associated with disease progression and prognosis. ResolvinD1 (RvD1) is a promising lipid mediator with critical protection against infection in the lung. However, whether RvD1 protects against IAV-induced injury and the underlying mechanisms remain elusive. In this study, primary normal human bronchial epithelial (pNHBE) cells were isolated and co-cultured with IAV and/or RvD1. Then, the expressions of E-cadherin, Zonula occludins-1, inflammatory mediators and proteins in Nrf2-dependent pathway were detected. To further explore the mechanisms, Nrf2 short hairpin RNA (Nrf2 shRNA) was applied in pNHBE cells. Furthermore, mice were infected with IAV, and were subsequently treated with RvD1. We found that IAV downregulated expressions of E-cadherin, Zonula occludins-1, Nrf2 and HO-1, upregulated the phosphorylation of NF κ B p65 and IKBα, levels of IL-8 and TNF-α, as well as ROS production. RvD1 reversed these damaging effects induced by IAV. However, when Nrf2 expression was suppressed with shRNA in pNHBE cells, the protective effects of RvD1 on IAV-induced injury were inhibited. In vivo studies further demonstrated that RvD1 could alleviate barrier protein breakdown and reduce airway inflammatory reactions. Collectively, the study demonstrated that RvD1 could play dual beneficial roles in protecting airway epithelium barrier function and reducing inflammation *via* the Nrf2 pathway, which may provide a better treatment option for influenza A virus infection.

## Introduction

Influenza A virus (IAV) can cause seasonal burden and even pandemics, leading to considerable morbidity and mortality throughout the world. As the front line, bronchial epithelial cells play an important role in defense against IAV (Sanders et al., 2011; Benam et al., 2019; LeMessurier et al., 2020). When the epithelial barrier is damaged, the risk of tissue penetration by microbes, toxins and allergens increases, as well as the succeeding inflammatory reaction (Wang et al., 2019). Clinical and experimental evidence suggested that epithelial injury and excessive host inflammation increased the risk of complications, such as severe respiratory insufficiency or secondary bacterial infection, in severe influenza virus infection, which was considered to be an important cause for the high mortality during the influenza pandemic (Kash and Taubenberger, 2015; Hebert et al., 2020).

The apical junction complex (AJC) is the main component of the epithelial physical barrier, it is composed of peripheral membrane molecules (such as zonula occludins-1, ZO-1) and adhesion junction molecules (such as E-cadherin, E-cad). The formation of AJC at the intercellular contact sites ensures the integrity of the barrier while regulates paracellular permeability. Damages to AJC connecting adjacent epithelial cells are considered to be important causes of barrier dysfunction (Uiprasertkul et al., 2007; Mauad et al., 2010; Short et al., 2016). However, whether and how IAV causes damages to the AJC of bronchial epithelial cells remain unclear.

It is reported that many factors can affect the AJC, such as LPS (Roxas et al., 2010; Peerapen and Thongboonkerd, 2011), cytokines (Capaldo and Nusrat, 2009), and ROS (Schreibelt et al., 2007). In the pathological process of influenza virus infection, a large number of inflammatory cytokines and ROS can be found (To et al., 2020). It was demonstrated that nuclear factor erythroid 2-related factor 2 (Nrf2) was critical for the protective effects on pulmonary inflammation and injury induced by IAV through activating antioxidant genes (Kosmider et al., 2012). Based on these findings, the Nrf2 pathway may play a key role in influenza virus infection.

ResolvinD1 (RvD1), a lipid mediator derived from omega-3 polyunsaturated fatty acid, displays anti-inflammatory and anti-oxidative activities (Spite et al., 2009; Eickmeier et al., 2013). Clinical and basic researches have indicated that RvD1 exhibits anti-inflammatory and immunomodulatory functions against pneumonia (Hsiao et al., 2014; Wang et al., 2017; Sham et al., 2018), acute lung injury (Wang et al., 2011) and ischemia/reperfusion injury (Zhao et al., 2016). Additionally, RvD1 has been reported to protect the integrity and barrier function of endothelial adherent junction from destruction induced by inflammatory mediators by inhibiting ROS production and preventing SHP2 inactivation (Chattopadhyay et al., 2017). Therefore, we make a hypothesis that RvD1 can protect the airway barrier and inhibit inflammation and provide a potential therapeutic option for influenza A virus infection.

In summary, the study aims to investigate whether RvD1 can protect against IAV-induced lung epithelial injury and inflammation *via* the activation of the Nrf2-dependent antioxidant pathway.

## Materials and Methods

### Ethics Statement

All experiments were conducted with the approval of the Biomedical and the Animal Ethics Committee of Anhui Medical University (NO.20180388; NO.20180430) and in strict accordance with ethical principles. All participants were informed the purpose of this study and agreed to written consent.

### Reagents and Materials

ResolvinD1 (17(S)-RvD1) was purchased from Cayman Chemical Company (USA). 2′,7′-dichlorofluorescein diacetate (DCFH-DA) were from Beyotime Corporation (Shanghai, China). Bronchial epithelial cell growth medium (BEGM, CC-3170) was purchased from Lonza (USA). Antibodies to E-cadherin (24E10), Phospho-NF-kB p65 (Ser536) (93H1), histone H3 (D1H2), NF-kB p65 (D14E12), and β-actin (13E5) were from Cell Signaling Technology (MA, USA), antibodies to ZO-1 (ab96587), HO-1 (ab13248), and Nrf2 (ab137550) were purchased from Abcam (USA), IKBα (WL01936) and p-IKBα (WL02495) were from Wanleibio Company (Shenyang, China). The transfection reagents for Nrf2 were from Hanbio (Shanghai, China).

### Cell Isolation and Culture

The primary normal human bronchial epithelial (pNHBE) cells were harvested from the bronchial tissues resected from patients with lung carcinoma *in situ* judged by two senior pathologists, the bronchial tissues were cut at the site more than 2 cm distant from the edge of lung carcinoma according to the method modified from previous studies (Fulcher et al., 2005; Yamaya et al., 2011). Bronchial tissues were collected, rinsed with PBS for three times and then cut into pieces, incubated overnight at 4°C in a solution of 100 μg/ml pronase and 1 μg/ml deoxyribonuclease, then further digested in another digestive fluid for 1 h, which contained ethylene diamine tetraacetic acid (EDTA, 2 nM), CaCl2 (0.75 mg/ml), MgCl2 (1 mg/ml), dl-dithiothreitol(0.05 mg/ml), collagenase (0.25 mg/ml), and deoxyribonuclease (10 μg/ml). After termination of digestion, filtration, and centrifugation at 500*g* for 5 min, pNHBE cells were obtained from the mixture and placed on a collagen-coated dish in BEGM medium. pNHBE cells were identified, cultured, and medium was changed every 2 days, then different experiments were conducted (Ji et al., 2020).

### Infection and Treatment of Cells

pNHBE cells were expanded on six-well dishes (3.0 × 10^5^ cells/well). When 70% to 80% confluence was reached, the medium containing hydrocortisone was removed and replaced with basal epithelial cell medium. At least 24 h later, pNHBE cells were infected with IAV/H3N2 (MOI 50). Subsequently, media was aspirated after 4 h. The cells were then treated with 200 nM of RvD1 in basal epithelial cell medium. RNA was extracted with Trizol, protein lysates were collected with RIPA lysis buffer, and immunofluorescence was analyzed after fixing with 4% paraformaldehyde (PFA).

### Western Blotting

To measure the expressions of Phospho-NF-kB p65, Nrf2, HO-1, ZO-1, E-cad, IKBα, p-IKBα, and NF-kB p65, total proteins were collected with RIPA lysis buffer and Nrf2 in cytosol and nucleus was also extracted. To extract cytoplasmic and nuclear solution, the cells were scraped off with the cell scraper. Cell pellet was collected by centrifugation, and 200 μl cytoplasmic protein extraction reagent A containing PMSF was added, then the mixture was vortex shocked for 5 s to make the cell pellet completely suspended. After ice bath for 10 min, 10 μl cytoplasmic protein extraction reagent B was added. Subsequently, the mixture was centrifuged at 12,000*g* 4°C for 5 min to get the supernatant as the extracted cytoplasmic protein. The remaining supernatant was completely absorbed and 50 μl nuclear protein extraction reagent was added. After ice bath and vortex shock for 20 s every 1 min for 30 min, the mixture was centrifuged at 12,000*g* 4°C for 10 min to obtain the supernatant as the extracted nuclear protein. Then proteins were quantified using BCA protein quantification kit and examined using Western blots analysis essentially as we described previously (Wu et al., 2015). The membranes were detected with enhanced chemo-luminescence reagent (ECL Advance, Amersham, UK), and the blots were quantified using densitometry analysis.

### Immunofluorescence

To examine cellular E-cad and Nrf2, the cells were fixed with 4% PFA for 10 min and permeabilized with 0.2%Triton-X-100 for 5 min. After blocking with 5% BSA for 1 h, the cells were stained with respective primary antibodies overnight at 4°C, and then incubated with the appropriate secondary antibody at room temperature for 90 min. DAPI staining was done for 5 min, the images were obtained with confocal microscopy.

### Quantitative Real-Time PCR

RNA was extracted and reverse-transcribed to cDNA according to kit procedures. Real-time PCR was performed with the SYBR Premix Ex Taq II (Tli RNaseH Plus) (Takara Biotechnology, Dalian, China). Expression levels of target gene were calculated through 2^−ΔΔ^Ct relative to the reference gene (β-actin), then calculated fold change relative to the media control. The following primers were used: Ecad (human) forward: 5′-AGTCACTGACACCAACGATAAT-3′, reverse: 5′-ATCGTTGTTCACTGGATTTGTG-3′; ZO-1 (human) forward: 5′-AAAGAGAAAGGTGAAACACTGC-3′, reverse: 5′-TTTTAGAGCAAAAGACCAACCG-3′; IL-8 (human) forward: 5′-AACTGAGAGTGATTGAGAGTGG-3′, reverse: 5′-ATGAATTCTCAGCCCTCTTCAA-3′; β-actin (human) forward: 5′−CCTGG CACCCAGCAC AAT-3′, reverse: 5′-GGGCCGGACTCGTCATAC-3′; TNF-α (human) forward: ATGTCTCAGCCTCTTCTCATTC, reverse: GCTTGTCACTCGAATTTTGAGA.

### Measurement of Intracellular ROS In Vitro

ROS was detected with the DCFH-DA. pNHBE cells were seeded and cultured on six-well cell culture plates, followed by the infection with H3N2 or treatment with RvD1 as described above. Then, 1 ml DCFH-DA (10 μm) was added to the plates and incubated for 20 min at 37°C. Subsequently, the cells were washed with pre-warmed PBS for three times to fully remove DCFH-DA that did not enter the cells. The excitation wavelength of 488 nm and emission wavelength of 530 nm were used to detect the intensity of fluorescence with a fully-automatic imaging system.

Cells were collected, and resuspended in PBS, and fluorescence intensity was measured by flow cytometry with the CytoFlex (Beckman Coulter, CA, USA). The excitation wavelength of 488 nm and emission wavelength of 530 nm were used to obtain the average fluorescence intensity from 10,000 cells using a band-pass filter.

### Lentiviral Vector Construction, Production, and Infection

Targeted oligonucleotides (5′-AGTTTGGGAGGAGCTATTATC-3′) were designed and cloned in pHBLV-U6-MCS-EF1-t2a-puro lentivirus RNAi vector (Hanbio, Shanghai, China). Then 293T cells were transfected with plasmid PSPAX2, PMD2G, and LipoFiter (Hanbio, Shanghai, China) to synthesize recombinant lentivirus. Supernatants containing lentivirus were collected 48 h after transfection, and filtered through 0.22-μm cellulose acetate filters (Millipore, USA). Then, the recombinant lentivirus was concentrated by super-centrifugation at a speed of 50,000*g* for 2 h.

In the knockdown experiment, HBE cells (0.5 × 10^6^ cells/well) were cultured on 6-well plates. When HBE cells reached 60% confluence, the cells were treated with lentivirus (MOI 50) combined with polybrene (5 μg/ml) for 24 h, then replace the medium containing virus with fresh medium to the HBE cells. Most HBE cells (80%) were found to express EGFP 48 h after transfection. The negative control was the empty lentivirus vector lenti-EGFP. HBE cells were collected 3 days after virus infection and the Western blot analysis was performed to examine the expression of Nrf2.

### Plaque Assay

Influenza A/Anhui/1/2017 (H3N2) virus, provided by Professor Liu Yan (Department of Microbiology, Anhui Medical University), was isolated from the patient in 2017 and used in laboratory studies under approved standard biosafety procedures. The H3N2 virus was amplified in Madin-Darby canine kidney (MDCK) cells and specific pathogen-free embryonated chicken eggs, and its titer was determined by standard plaque assay on MDCK cells. Virus or lung tissue homogenate continuously diluted with 1% bovine serum albumin DMEM was used to infect single MDCK cells at 37°C for 2 h. After washed with PBS, cultivation was continued with 50% 2× DMEM, 50% avecil (2.35%) and N-acetyl trypsin (1.5 μg/ml) for 72 h. The medium was removed, the monolayer was stained with naphthalene blue-black and plaques were then counted. All experiments involving virus were conducted in accordance with biosafety level II requirements and personal protective equipment was recommended for all participating researchers.

### Animal Experiment

BALB/c mice (18–22 g, 6–8 weeks) were taken from the animal services unit of Anhui Medical University. They were given a standard laboratory diet and placed under specific conditions that the temperatures ranged from 20°C to 25°C with 12 h light/dark cycle.

Forty mice were randomly divided into four groups: control group, H3N2 group, H3N2 + RvD1 group, and RvD1 group. Mice were anesthetized with pentobarbital (70 mg/Kg), then received 100 μl sterile PBS or infected with 100 PFU of H3N2 in 100 μl through the oropharyngeal aspiration (Abood et al., 2019; Hebert et al., 2020). The H3N2 + RvD1 group and the RvD1 group received RvD1 100 ng intravenously on days 4 and 6, respectively.

### Bronchoalveolar Lavage

Mice were euthanized with pentobarbital intraperitoneal injection after the treatment with PBS or IAV for 7 days. Then the trachea and lung tissues were dissected gently, and a syringe needle was inserted into the trachea and fixed. After the right main bronchus was ligated, the left lung was rinsed with 1 ml cold PBS for three times to collect the BALF, then centrifugation at 700*g* 4°C for 10 min, the supernatant was collected for subsequent tests. The pellet was resuspended, and the cells were counted with a hemocytometer. BALF cells were further classified and counted after Wright-Giemsa staining. The right lung was harvested for morphological analysis.

### Morphologic Analysis

The right Lungs were harvested and fixed with 4% PFA for at least 24 h. Following dehydration and paraffin embedding, the lungs were sectioned in 5 μm. Then hematoxylin and eosin staining were used to assess inflammatory cell infiltration. Semi-quantitative scoring for HE staining specimens was conducted according to the methods in previous study (Hebert et al., 2020). The expression of E-cad was detected by Immunohistochemical staining with primary antibody against-E-cad (1:200).

### Measurement of Bronchial Epithelial Permeability

FITC-Dextran (molecular weight 4000 Da; FD4) is a marker consisting of coupling fluorescein-isothiocyanate to dextran, which can be used to determine permeability of the barrier based on the size of the dextran used. After H3N2 challenge for 7 days, the mice were injected intravenously (i.v.) with FD4 (25 mg/ml). Then, the animals were anesthetized after 10 min. BALF was collected as described above with 1 ml PBS for three times. Blood was collected *via* heart puncture, then centrifuged to obtain the serum. BALF and serum were diluted with PBS and their fluorescence values were detected at 515 nm. The permeability of lung epithelial was calculated using the fluorescence ratio of BALF to serum.

### ELISA

According to the instructions, the concentration of IL-8 (Absin Bioscience, Shanghai, China ) in BALF was quantified, as well as TNF-α using ELISA kits (Dakewe Biological Technology, Beijing, China).

### Statistical Analysis

Continuous variables with normal distribution were presented as mean ± standard deviation (SD). *t*-test or One-way ANOVA was used to determine significance with SPSS17.0 and GraphPad 5 software when comparing two groups or more groups. Statistically significant were indicated as *P* < 0.05.

## Results

### Effects of IAV and RvD1 on the Expressions of E-cad and ZO-1 in pNHBE Cells

**Figure 1:**
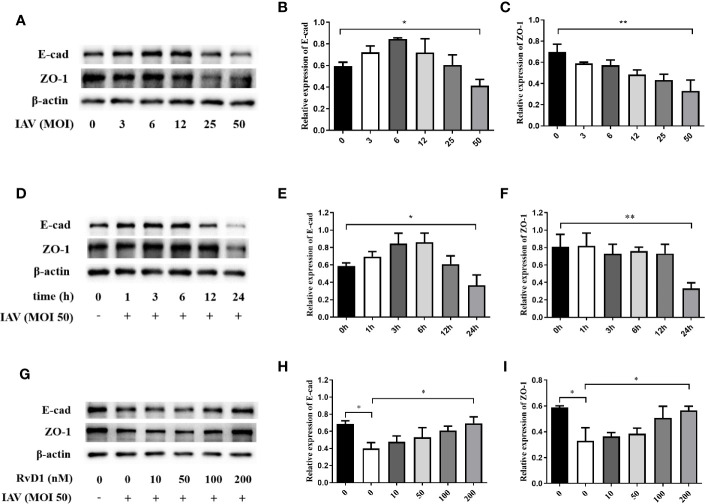
Effects of IAV and RvD1 on the expressions of E-cad and ZO-1 in pNHBE cells. After the infection of pNHBE cells with different concentration of IAV for 24 h, the expressions of E-cad and ZO-1 were detected by Western blotting **(A–C)**. After the infection of p-NHBE cells with IAV (MOI 50) for different times, the expressions of E-cad and ZO-1 were measured by Western blotting **(D–F)**. Different concentration of RvD1 were administered with IAV infection for 24 h in pNHBE cells, and the expressions of E-cad and ZO-1 were evaluated by Western blotting **(G–I)**. Quantification of the expressions of E-cad and ZO-1 as referencing to β-actin (^*^P < 0.05, ^**^P < 0.01). Each dataset comprises three independent experiments.

To detect the optimal action concentration and time of IAV, p-NHBE cells were infected with IAV (MOI 0, 3, 6, 12, 25, or 50) for 24 h, and infected with IAV (MOI 50) for 1, 3, 6, 12, or 24 h, the results showed that the inhibition of E-cad and ZO-1 expression was the most obvious 24 h after IAV (MOI 50) infection (P < 0.05; **Figure 1**). Furthermore, to determine the optimal concentration of RvD1, pNHBE cells were treated with RvD1 at 0, 10, 50, 100, or 200 nmol/l; it was found that E-cad and ZO-1 were most protected at 200 nmol/l in the presence of IAV for 24 h. Therefore, all further *in vitro* experiments were performed using 200 nmol/l of RvD1 and IAV (MOI 50) for 24 h.

### RvD1 Protected Bronchial Epithelial Cells From Damage Induced by H3N2

**Figure 2:**
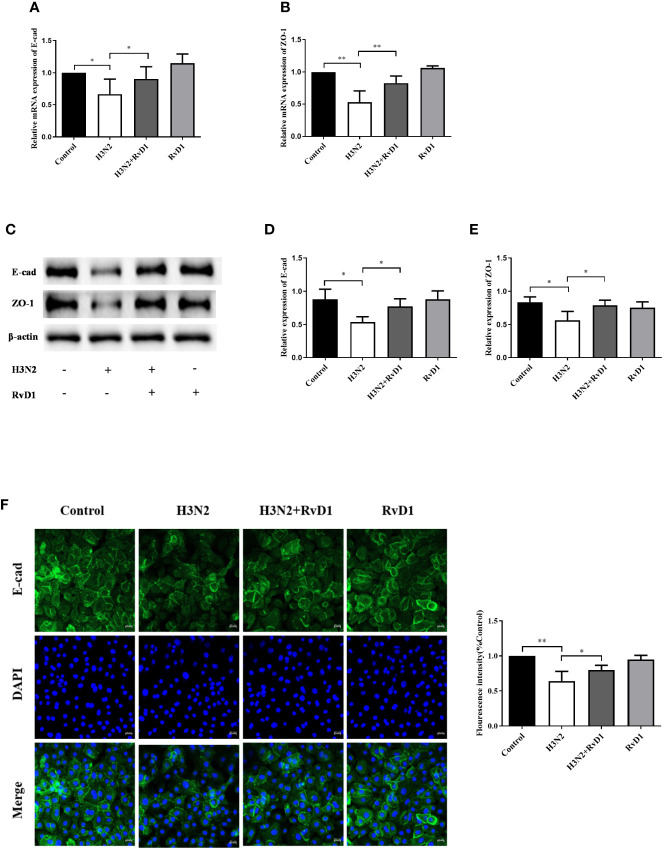
RvD1 mediated protection against the damage of E-cad and ZO-1 in airway epithelial cells induced by IAV. pNHBE cells were infected with IAV (MOI 50) and/or treated with 200 nM RvD1 for 24 h, qRT-PCR was performed to detect the mRNA levels of E-cad and ZO-1 **(A, B)** and the expressions of them were detected by western blotting **(C–E)**. Immunofluorescence staining was performed to detect the expression of E-cad by staining E-cad (green) and DAPI (blue) **(F)**. E-cad and ZO-1 were normalized to β-actin. Each dataset comprises three independent experiments (^*^P < 0.05, ^**^P < 0.01).

We investigated the effects of H3N2 and/or RvD1 on the expressions of E-cad and ZO-1 in the pNHBE cells. In **Figure 2**, the mRNA and protein expression levels of E-cad and ZO-1 in the H3N2-infected cells were lower compared with those in controls (P < 0.05), and RvD1 treatment significantly prevented these reductions (P < 0.05). The effects of RvD1 were further confirmed by immunofluorescence staining. As demonstrated in **Figure 2**, E-cad staining in the cell membranes of the control was prominent and complete. Following H3N2 infection, the E-cad staining around the cells was significantly weakened and incomplete; similarly, RvD1 effectively prevented those changes.

### RvD1 Inhibited Inflammation Induced by H3N2

**Figure 3:**
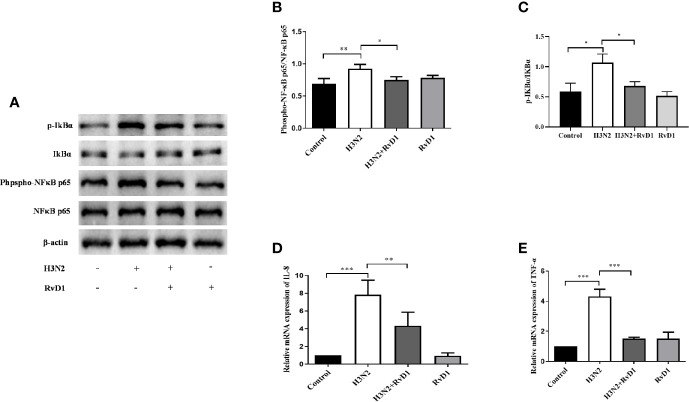
RvD1 inhibited inflammation induced by H3N2 in airway epithelial cells. pNHBE cells were infected with IAV (MOI 50) and/or treated with 200 nM RvD1 for 24 h. Western blotting was performed to evaluate the expressions of Phospho-NF-kB p65, NF-kB p65, IKBα and p-IKBα **(A)**. Phospho-NF-kB p65 was normalized to NF-kB p65 **(B)**. p-IKBα was normalized to IKBα **(C)**. qRT-PCR was performed to detect the mRNA levels of IL-8 and TNF-α **(D, E)**. The results shown were from three separate experiments (^*^P < 0.05, ^**^P < 0.01, ^***^P < 0.001).

To investigate whether RvD1 inhibited bronchial inflammation, we measured and compared the expressions of Phospho-NF κ B p65, NF κ B p65, IKBα, and p-IKBα in pNHBE cells. As shown in **Figure 3**, IAV infection promoted NF κ B p65 and IKBα phosphorylation, stimulated IL-8 and TNF-α mRNA expressions, and RvD1 effectively inhibited these responses (P < 0.05).

### RvD1 Blocked the H3N2-Induced ROS Production

**Figure 4:**
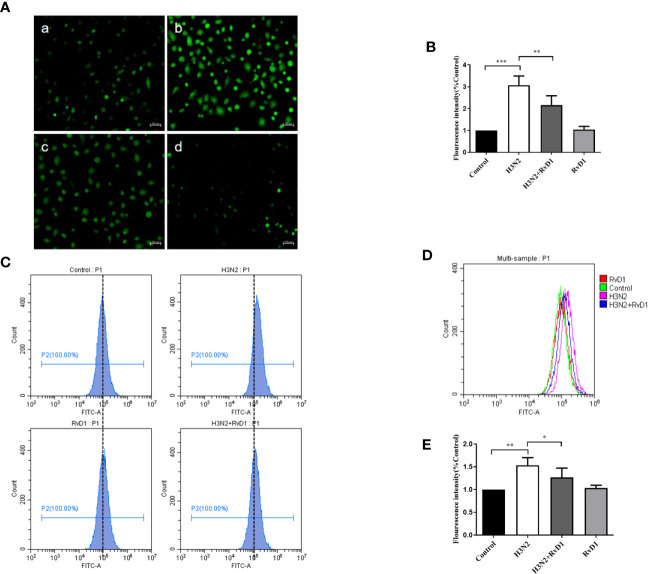
RvD1 inhibited the ROS production induced by IAV. pNHBE cells were processed with IAV (MOI 50) and/or 200 nM RvD1 for 24 h. DCFH-DA was used to determine the cellular ROS in Control group **(Aa)**, H3N2 group **(Ab)**, H3N2+RvD1 group **(Ac)** and RvD1 group **(Ad)**. Data were presented as the fluorescence values over control group **(B)**. Fluorescence intensity was also measured by flow cytometry, the results were presented in histogram as the fluorescence values over control group **(C–E)**. Each dataset comprises three independent experiments (^*^P < 0.05, ^**^P < 0.01, ^***^P < 0.001).

Considering that excessive ROS can mediate the destruction of cell connections, we investigated whether ROS production was increased in bronchial epithelial cell in H3N2 infection, and also examined the role of RvD1 in this process. In **Figure 4**, the DCF fluorescence intensity of pNHBE cells increased after infected with H3N2 (MOI 50) for 24 h, indicating that the intracellular ROS levels induced by H3N2 were higher compared with the control group. RvD1 (200 nM) significantly inhibited these effects of H3N2 (P < 0.05).

### RvD1 Promoted the Activation of Nrf2 in HBE Cells

**Figure 5:**
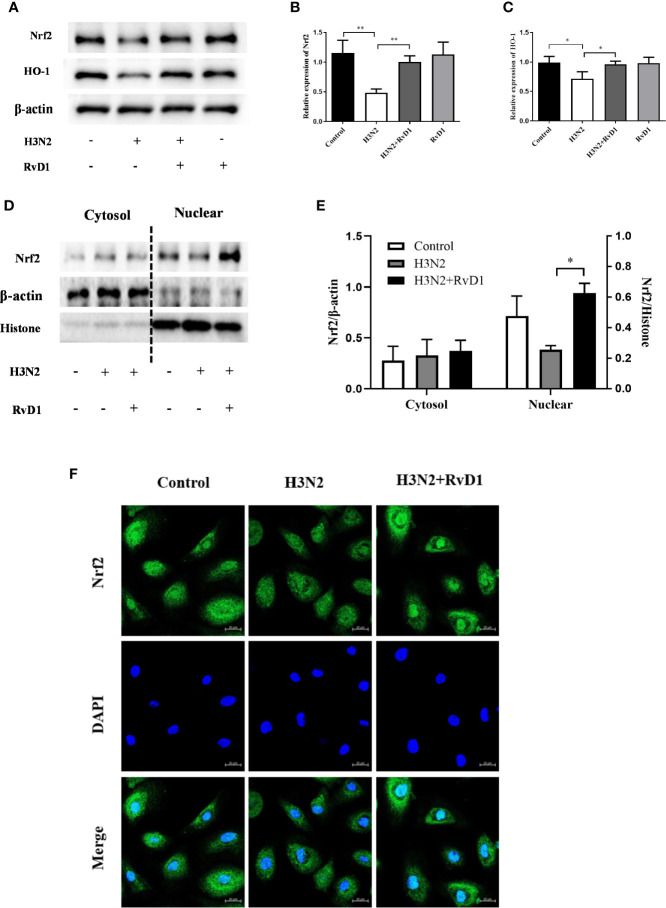
RvD1 promoted the activities of Nrf2 in pNHBE cells. pNHBE cells were infected with IAV (MOI 50) alone or in combination with 200 nM RvD1 for 24 h. Western blotting was performed to measure the protein levels of Nrf2 and HO-1 **(A)**. Quantification of the expressions of Nrf2 and HO-1 as referencing to β-actin **(B, C)**. Nrf2 protein levels in cytoplasm or nuclei were measured using Western blot analysis **(D)**. Quantification of the expressions of Nrf2 in cytoplasm and nuclei as referencing to β-actin and histone, respectively **(E)**. Immunofluorescence staining and confocal imaging was performed to detect the expression of Nrf2 by staining Nrf2 (green) and DAPI (blue) **(F)** (^*^P < 0.05, ^**^P < 0.01. The scale bar indicates 20 μm).

Nrf2 is a major antioxidant factor, which can activate the production of antioxidant factors such as HO-1, thus reducing the production of ROS. After confirming that RvD1 can inhibit intracellular ROS accumulation in pNHBE cells after H3N2 infection, we compared the expressions of Nrf2 under different experimental conditions. **Figure 5A** shows H3N2 infection markedly inhibited Nrf2 expression in HBE cells, and this inhibition was prevented by RvD1. HO-1, a classic downstream gene of Nrf2, has a variety of beneficial activities, including clearing toxic heme and preventing oxidative stress and inflammation. Similar to the effect on Nrf2, RvD1 also significantly increased the expression of HO-1.

Nrf2 is a nuclear transcription factor. To further investigate whether RvD1 plays a role in Nrf2 nuclear translocation, protein levels of Nrf2 in cytoplasmic and nuclear extracts were evaluated with western blot analysis. The expressions of Nrf2 in nucleus were improved after the treatment of RvD1, suggesting that RvD1 promoted the translocation of Nrf2 to nucleus. These results were coincident with immunofluorescence (**Figure 5**). Our results confirmed that H3N2 infection led to a substantially decreased in Nrf2 and HO-1 expression, while RvD1 reversed such an effect and induced the nuclear translocation of Nrf2.

### Nrf2 Mediated the Effect of RvD1 on pNHBE Cells

**Figure 6:**
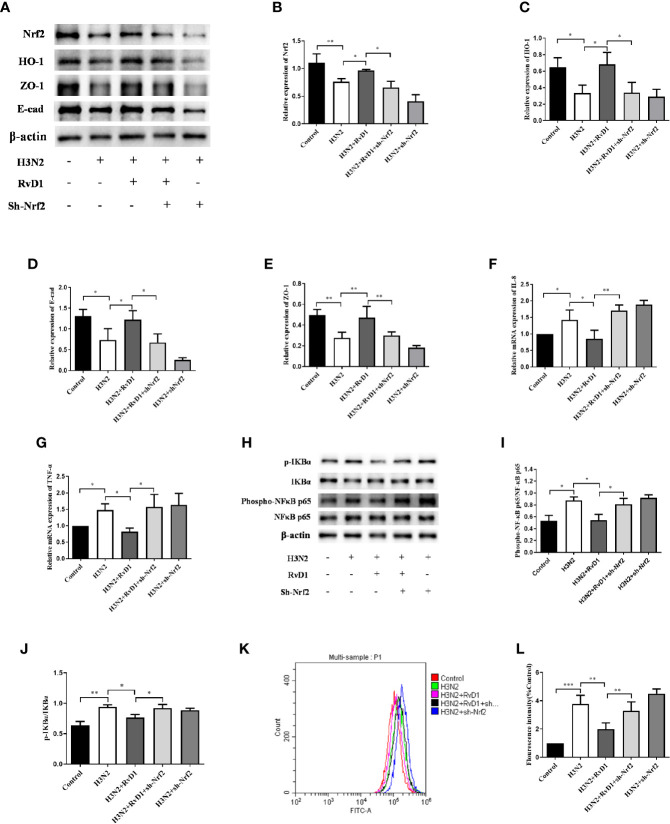
RvD1 exerted the protective effects against IAV-induced injury required Nrf2. pNHBE cells were transfected with or without shNrf2, then cells were infected with IAV (MOI 50) for 24 h with or without 200 nM RvD1. The total proteins of cell lysis were analyzed by Western blotting. Densitometry was used to analyze the blots, and quantification of the expressions as referencing to β-actin **(A–E)**, Phospho-NF-kB p65 was normalized to NF-kB p65 **(H, I)**, p-IKBα was normalized to IKBα **(H, J)**. The mRNA levels of IL-8 and TNF- α were detected by RT-PCR **(F, G)**. Measured cellular ROS with the use of DCFH-DA, the results in histogram were showed as the ratio of fluorescence values in different stimulus groups to those in the control cells **(K, L)**. (^*^P < 0.05, ^**^P < 0.05, ^***^ P < 0.001). Each dataset comprises three independent experiments.

To investigate whether Nrf2 was involved in the effects of RvD1 on the barrier and inflammation-related protein expressions, we transfected pNHBE cells with recombinant lentiviruses HBLV-H-Nrf2 shRNA-PURO, followed by the intervention of IAV and/or RvD1. As demonstrated in **Figure 6**, RvD1 inhibited the destructive effects of H3N2 on E-cad and ZO-1 expressions, as well as that on the levels of IL-8 and TNF-α. However, when IAV-infected cells were transfected with sh-Nrf2 simultaneously, the effect of RvD1 on E-cad and ZO-1 expressions was diminished; and concomitantly, a substantially increased production of ROS, expressions of Phospho-NF κ B p65, p-IKBα, levels of IL-8 and TNF-α were observed. This suggested that the effect of RvD1 on HBE cells induced by IAV possibly occur through the Nrf2 pathway.

### RvD1 Alleviated Damage in IAV-Infected Mice

**Figure 7:**
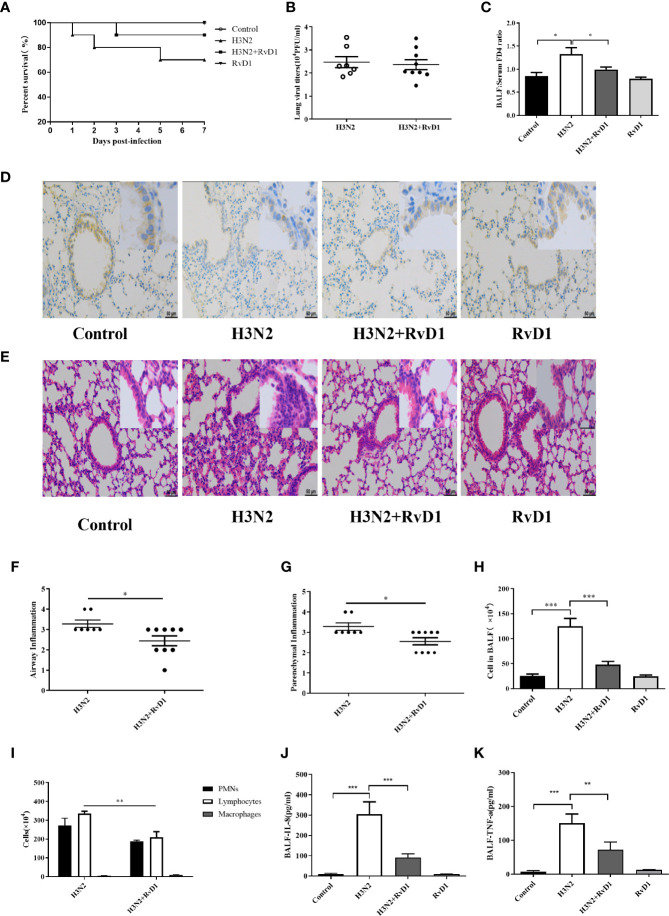
RvD1 alleviated damage in IAV-infected mice. After the treatment with 0.9% saline or 100 μl IAV in saline *via* oropharyngeal aspiration, RvD1 (100 ng) was administered i.v. 4 and 6 days later in mice. On day 7, mice were sacrificed under final anesthesia. BALF and lung tissue were harvested for cell count and morphological analysis. Survival rates were shown **(A)**. Lung viral load was measured **(B)**. 7 days after inhaling 0.9% saline or IAV, mice were injected with FD4. As before, collected BALF and serum, and determined the FD4 ratio of BALF to serum **(C)**. Immunohistochemical was performed to localize the E-cad in bronchial epithelium. Scale bars = 50 μm **(D)**. H&E staining and the semi-quantitative scoring of inflammation in lung tissue **(E–G)**. Total cell counts and differential counts in BALF **(H, I)**. The levels of IL-8 and TNF-α in BALF **(J, K)** (^*^P < 0.05, ^**^P < 0.01,^***^P < 0.001).

To investigate the *in vivo* effects of RvD1, mice with IAV infection were established and analyzed. In general, H3N2 caused a significant inflammatory response 7 days after infection, and most of cytokines and chemokines in BALF were found to peak at 6 to 7 days after bronchial H3N2 inhalation in mice, accompanied by neutrophil and lymphocytes infiltration (Abood et al., 2019; Hebert et al., 2020). Therefore, all changes *in vivo* were analyzed 7 days after H3N2 infection. BALF was collected and lung histological analysis was performed. As described in **Figure 7** and **Supplementary Figure 1**, the bronchial and alveolar spaces of mice in the H3N2 group were obviously infiltrated by inflammatory cells, neutrophils, lymphocytes and cytokines in BALF were also significantly increased, which was similar to our previous reports (Ji et al., 2020). RvD1 accelerated the resolution of inflammation when administered intravenously 4 and 6 days after inhalation of H3N2. Neutrophils, lymphocytes and cytokines in BALF were significantly reduced after the treatment with RvD1 (P < 0.05). However, supplement with RvD1 had no significant effect on the inhibition of viral proliferation. Despite it seemed to reduce mortality, but there was no significant difference.

Also, RvD1 had a protective effect against H3N2-induced airway injury; as shown in **Figure 7**, we could clearly see that bronchial epithelial cells in mice were damaged after H3N2 challenge, and staining of E-cad was weak as compared with that in control mice. RvD1 apparently inhibited such epithelial destruction. Moreover, changes in barrier function were further observed by detecting FD4 leakage after intervention with H3N2 and/or RvD1. The FD4 ratio of BALF to serum rise from 0.743 to 1.371(P < 0.05). With RvD1, the ratio drops to 0.951(P<0.05), suggesting that RvD1 effectively protects bronchial epithelium from H3N2-induced permeability changes (**Figure 7**). Thus, changes in barrier function were further understood by examining the effects of H3N2 and/or RvD1 on FD4 leakage.

## Discussion

IAV is the most important human pathogen, causing substantial seasonal and pandemic morbidity and mortality (Short et al., 2014). IAV targets airway epithelial cells, which were essential for maintaining respiratory homeostasis and preventing the lungs from the invasion of harmful environmental substances (Slepushkin et al., 2001; Förster, 2008). When the epithelial barrier is damaged, excessive inflammation, exudation of fluid and protein, and secondary bacterial infection may lead to serious complications during influenza virus infection (Abed et al., 2002; Guttman and Finlay, 2009; Chen et al., 2019). It is suggested that the injury of the airway barrier and uncontrolled inflammatory response are closely related to the prognosis and progression of influenza A virus infection. Therefore, it is essential to prevent severe influenza events by enhancing the airway integrity and pro-inflammatory regression during IAV infection; however, few studies have been conducted.

To investigate the changes in bronchial epithelial barrier during IAV infection, we detected the main components of the barrier, including the adherent junction protein (E-cad) and tight junction protein (ZO-1), which played vital roles in regulating paracellular permeability and maintaining epithelial cells barrier function. Also, *in vitro* we employed primary NHBE cells, separated from the normal human airway retaining features of the native epithelium, they have unique advantages over other commonly used epithelial cell lines. The results of western blotting, RT-PCR, and immunofluorescence assays confirmed that IAV infection inhibited the expressions of E-cad and ZO-1 in pNHBE cells and increased levels of IL-8 and TNF-α. In vivo, We found that IAV infection significantly impaired epithelial integrity in mice, including decreased E-cad expression, increased airway permeability, and inflammatory exudation, which were manifested by more leakage of FD4 into BALF, aggregation of inflammatory cells in BALF and around bronchioles, and elevated inflammatory cytokines in BALF.

To demonstrate the role of RvD1 in IAV infection, we treated cells or mice with RvD1 after IAV infection. We found that RvD1 had a number of benefits in treating IAV infection. Overall, RvD1 inhibited barrier destruction and inflammation and showed similar effects at the mRNA and protein levels *in vitro*. In vivo RvD1 also showed a protective effect on the destruction of the barrier and uncontrolled inflammation induced by IAV, despite it had no significant effect on the inhibition of virus proliferation, there were differences in inflammation and exudation. Although survival rate improved, there was no significant differences, which might be related to sample size and the length of observation time. Consistent with our results, Hsiao HM demonstrated that RvD1 alleviated inflammatory in human bronchial epithelial cells induced by polyinosinic-polycytidylic acid through TAK1 (Hsiao et al., 2014). Previous research showed that the ratio of RvD1 relative to SAA in serum was markedly reduced in IAV-infected mice on day 7, and RvD1 reduced lung infection and inflammation in the influenza virus and bacterial coinfection pneumonia model (Wang et al., 2017). Machado, F.S. also found that greater influenza A virus virulence may be associated with the loss of lipoxin’s pro-resolution effects, suggesting a protective role for lipoxin in this infection, which is an analogue of RvD1 (Cilloniz et al., 2010). In addition, it was demonstrated that RvD1 reverted the disruption of TJ protein and the increase of cellular permeability in human vascular endothelial cells induced by LPS through regulating IκBα signaling (Zhang et al., 2013). Therefore, it can be expected that RvD1 may represent an advancement in theoretical and clinical application for IAV infection.

To further explore the mechanism, we examined the levels of oxidative stress in cells. Overproduction of ROS during influenza A virus infection is a key contributor to lung injury (Akaike et al., 1996; Imai et al., 2008), and it is also one of the causes of junction protein injury. Nrf2, a basic regional leucine zipper transcription factor, can induce the expression of antioxidant enzymes and stage II enzymes such as glutathione S-transferase and NQO147, thereby protecting cells and tissues from oxidative stress (Keum and Choi, 2014; Al-Sawaf et al., 2015). It was suggested that Nrf2 increased the expression of E-cad to enhance the barrier function of airway epithelial (Sussan et al., 2015). Furthermore, HO-1, target gene of Nrf2, is reported to be closely related to E-cad under different conditions. Previous studies also highlighted the crucial requirements of HO-1-HMGB1 pathway for RvD1 in protecting against lung injury induced by mechanical ventilation (Sun et al., 2019). In the present study, our result showed that RvD1 could effectively inhibit the increase in ROS and inflammatory mediators in bronchial epithelial cells after H3N2 infection, in addition to upregulation of E-cad, ZO-1, Nrf2, and HO-1 in the bronchial epithelium. In addition, the results showed that Nrf2 pathway was essential for the upregulation of junction proteins and the regression of inflammation. When Nrf2 was knocked down by lentivirus in pNHBE cells, RvD1 had less effect on the expressions of E-cad, ZO-1, IL-8, and TNF-α. In summary, these results confirmed that Nrf2, involving in the protection of the airway barrier function, played a crucial part in the protection of RvD1 against airway injury induced by IAV.

Inflammatory cytokines are known to disrupt the tight junctions of airway epithelium to increase permeability (Capaldo and Nusrat, 2009). When the epithelial barrier was damaged, exudation of inflammation was increased. As a result, barrier dysfunction and inflammation create a self-perpetuating cycle, and blocking one is often not enough to ease the disease process. RvD1 has a unique advantage of promoting both barrier function recovery and inflammation regression. These data have implications not only for the treatment of IAV infection, but also for the prevention of secondary bacterial infection after influenza or other emerging viral respiratory infections, such as COVID-19.

**Figure 8:**
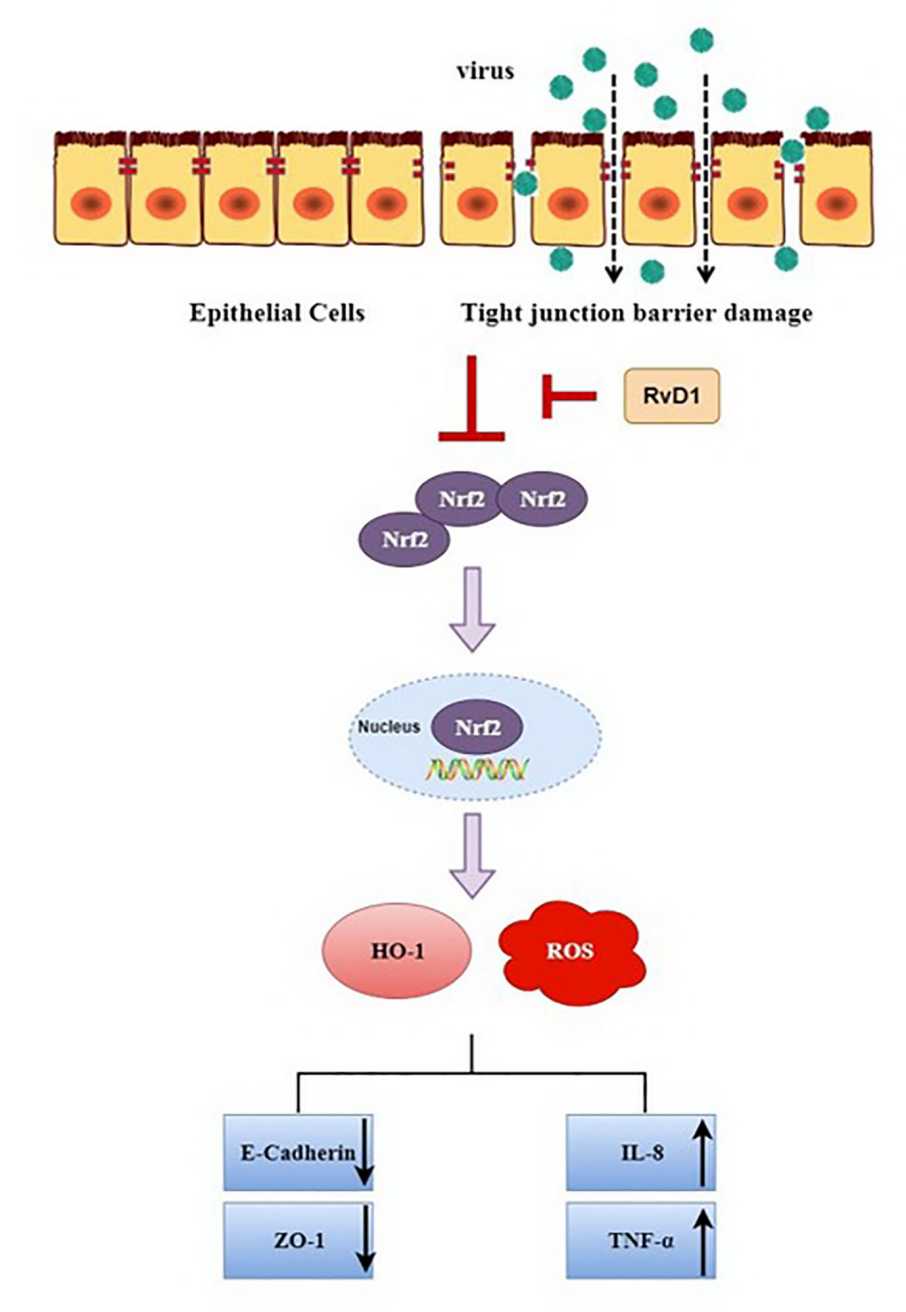
Potential mechanisms of RvD1 protecting airway epithelial cells from injury induced by IAV *via* Nrf2 pathway.

In conclusion, our present study demonstrated that after IAV infection, for the first time to our knowledge, ROS and inflammatory cytokines were markedly increased, while the expressions of E-cad, ZO-1, Nrf2, and HO-1 were significantly inhibited, these changes were reversed by RvD1. Also, these findings were detected *in vivo*; thus suggesting that RvD1 protects the airway barrier from injury induced by IAV through the Nrf2 pathway (**Figure 8**), and it may provide a better treatment option for influenza A virus infection.

## Data Availability Statement

The raw data supporting the conclusions of this article will be made available by the authors, without undue reservation.

## Ethics Statement

All experiments were conducted with the approval of the Biomedical and the Animal Ethics Committee of Anhui Medical University (NO.20180388; NO.20180430) and in strict accordance with ethical principles. All participants were informed the purpose of this study and agreed to written consent.

## Author Contributions

YG and Y-HT were the main investigator of the project and drafted the manuscript. XW and SJ contributed in performing the experiment and analyzing the data. J-LS provided a place for experiments. H-MW and G-HF were the corresponding authors who contributed in designing the research project, funding, and revising the manuscript. All authors contributed to the article and approved the submitted version.

## Funding

This work was funded by the the National Natural Science Foundation of China (Nos.81870036, 81770032) and the Key Technology Research and Development Program of Anhui Province (1804h08020237).

## Conflict of Interest

The authors declare that the research was conducted in the absence of any commercial or financial relationships that could be construed as a potential conflict of interest.

## References

[B1] Abed Y. Bourgault A. M. Fenton R. J. Morley P. J. Gower D. Owens I. J.. (2002). Characterization of 2 influenza A(H3N2) clinical isolates with reduced susceptibility to neuraminidase inhibitors due to mutations in the hemagglutinin gene. J. Infect. Dis. 186, 1074–1080. 10.1086/344237 12355356

[B2] Abood R. N. McHugh K. J. Rich H. E. Ortiz M. A. Tobin J. M. Ramanan K.. (2019). IL-22-binding protein exacerbates influenza, bacterial super-infection. Mucosal Immunol. 12, 1231–1243. 10.1038/s41385-019-0188-7 31296910 PMC6717528

[B3] Akaike T. Noguchi Y. Ijiri S. Setoguchi K. Suga M. Zheng Y. M.. (1996). Pathogenesis of influenza virus-induced pneumonia: involvement of both nitric oxide and oxygen radicals. Proc. Natl. Acad. Sci. U. S. A. 93, 2448–2453. 10.1073/pnas.93.6.2448 8637894 PMC39817

[B4] Al-Sawaf O. Clarner T. Fragoulis A. Kan Y. W. Pufe T. Streetz K.. (2015). Nrf2 in health and disease: current and future clinical implications. Clin. Sci. (Lond.) 129, 989–999. 10.1042/cs20150436 26386022

[B5] Benam K. H. Denney L. Ho L. P. (2019). How the Respiratory Epithelium Senses and Reacts to Influenza Virus. Am. J. Respir Cell Mol. Biol. 60, 259–268. 10.1165/rcmb.2018-0247TR 30372120 PMC6943877

[B6] Capaldo C. T. Nusrat A. (2009). Cytokine regulation of tight junctions. Biochim. Biophys. Acta 1788, 864–871. 10.1016/j.bbamem.2008.08.027 18952050 PMC2699410

[B7] Chattopadhyay R. Raghavan S. Rao G. N. (2017). Resolvin D1 via prevention of ROS-mediated SHP2 inactivation protects endothelial adherens junction integrity and barrier function. Redox Biol. 12, 438–455. 10.1016/j.redox.2017.02.023 28319894 PMC5357675

[B8] Chen Z. G. Wang Z. N. Yan Y. Liu J. He T. T. Thong K. T.. (2019). Upregulation of cell-surface mucin MUC15 in human nasal epithelial cells upon influenza A virus infection. BMC Infect. Dis. 19, 622. 10.1186/s12879-019-4213-y 31307416 PMC6631914

[B9] Cilloniz C. Pantin-Jackwood M. J. Ni C. Goodman A. G. Peng X. Proll S. C.. (2010). Lethal dissemination of H5N1 influenza virus is associated with dysregulation of inflammation and lipoxin signaling in a mouse model of infection. J. Virol 84, 7613–7624. 10.1128/jvi.00553-10 20504916 PMC2897611

[B10] Eickmeier O. Seki H. Haworth O. Hilberath J. N. Gao F. Uddin M.. (2013). Aspirin-triggered resolvin D1 reduces mucosal inflammation and promotes resolution in a murine model of acute lung injury. Mucosal Immunol. 6, 256–266. 10.1038/mi.2012.66 22785226 PMC3511650

[B11] Förster C. (2008). Tight junctions and the modulation of barrier function in disease. Histochem Cell Biol. 130, 55–70. 10.1007/s00418-008-0424-9 18415116 PMC2413111

[B12] Fulcher M. L. Gabriel S. Burns K. A. Yankaskas J. R. Randell S. H. (2005). Well-Differentiated Human Airway Epithelial Cell Cultures. Methods Mol. Med. 107, 183–206. 10.1385/1-59259-861-7:183 15492373

[B13] Guttman J. A. Finlay B. B. (2009). Tight junctions as targets of infectious agents. Biochim. Biophys. Acta 1788, 832–841. 10.1016/j.bbamem.2008.10.028 19059200

[B14] Hebert K. D. McLaughlin N. Galeas-Pena M. Zhang Z. Eddens T. Govero A.. (2020). Targeting the IL-22/IL-22BP axis enhances tight junctions and reduces inflammation during influenza infection. Mucosal Immunol. 13, 64–74. 10.1038/s41385-019-0206-9 31597930 PMC6917921

[B15] Hsiao H. M. Thatcher T. H. Levy E. P. Fulton R. A. Owens K. M. Phipps R. P.. (2014). Resolvin D1 attenuates polyinosinic-polycytidylic acid-induced inflammatory signaling in human airway epithelial cells via TAK1. J. Immunol. 193, 4980–4987. 10.4049/jimmunol.1400313 25320283 PMC4409010

[B16] Imai Y. Kuba K. Neely G. G. Yaghubian-Malhami R. Perkmann T. van Loo G.. (2008). Identification of oxidative stress and Toll-like receptor 4 signaling as a key pathway of acute lung injury. Cell 133, 235–249. 10.1016/j.cell.2008.02.043 18423196 PMC7112336

[B17] Ji S. Bai Q. Wu X. Zhang D. W. Wang S. Shen J. L.. (2020). Unique synergistic antiviral effects of Shufeng Jiedu Capsule and oseltamivir in influenza A viral-induced acute exacerbation of chronic obstructive pulmonary disease. BioMed. Pharmacother. 121, 109652. 10.1016/j.biopha.2019.109652 31734578

[B18] Kash J. C. Taubenberger J. K. (2015). The role of viral, host, and secondary bacterial factors in influenza pathogenesis. Am. J. Pathol. 185, 1528–1536. 10.1016/j.ajpath.2014.08.030 25747532 PMC4450310

[B19] Keum Y. S. Choi B. Y. (2014). Molecular and chemical regulation of the Keap1-Nrf2 signaling pathway. Molecules 19, 10074–10089. 10.3390/molecules190710074 25014534 PMC6270911

[B20] Kosmider B. Messier E. M. Janssen W. J. Nahreini P. Wang J. Hartshorn K. L.. (2012). Nrf2 protects human alveolar epithelial cells against injury induced by influenza A virus. Respir. Res. 13, 43. 10.1186/1465-9921-13-43 22672594 PMC3520784

[B21] LeMessurier K. S. Tiwary M. Morin N. P. Samarasinghe A. E. (2020). Respiratory Barrier as a Safeguard and Regulator of Defense Against Influenza A Virus and Streptococcus pneumoniae. Front. Immunol. 11, 3. 10.3389/fimmu.2020.00003 32117216 PMC7011736

[B22] Mauad T. Hajjar L. A. Callegari G. D. da Silva L. F. Schout D. Galas F. R.. (2010). Lung pathology in fatal novel human influenza A (H1N1) infection. Am. J. Respir. Crit. Care Med. 181, 72–79. 10.1164/rccm.200909-1420OC 19875682

[B23] Peerapen P. Thongboonkerd V. (2011). Effects of calcium oxalate monohydrate crystals on expression and function of tight junction of renal tubular epithelial cells. Lab. Invest. 91, 97–105. 10.1038/labinvest.2010.167 20856225

[B24] Roxas J. L. Koutsouris A. Bellmeyer A. Tesfay S. Royan S. Falzari K.. (2010). Enterohemorrhagic E. coli alters murine intestinal epithelial tight junction protein expression and barrier function in a Shiga toxin independent manner. Lab. Invest. 90, 1152–1168. 10.1038/labinvest.2010.91 20479715 PMC2912457

[B25] Sanders C. J. Doherty P. C. Thomas P. G. (2011). Respiratory epithelial cells in innate immunity to influenza virus infection. Cell Tissue Res. 343, 13–21. 10.1007/s00441-010-1043-z 20848130

[B26] Schreibelt G. Kooij G. Reijerkerk A. van Doorn R. Gringhuis S. I. van der Pol S.. (2007). Reactive oxygen species alter brain endothelial tight junction dynamics via RhoA, PI3 kinase, and PKB signaling. FASEB J. 21, 3666–3676. 10.1096/fj.07-8329com 17586731

[B27] Sham H. P. Walker K. H. Abdulnour R. E. Krishnamoorthy N. Douda D. N. Norris P. C.. (2018). 15-epi-Lipoxin A(4), Resolvin D2, and Resolvin D3 Induce NF-κB Regulators in Bacterial Pneumonia. J. Immunol. 200, 2757–2766. 10.4049/jimmunol.1602090 29523657 PMC5906795

[B28] Short K. R. Kroeze E. Fouchier R. A. M. Kuiken T. (2014). Pathogenesis of influenza-induced acute respiratory distress syndrome. Lancet Infect. Dis. 14, 57–69. 10.1016/s1473-3099(13)70286-x 24239327

[B29] Short K. R. Kasper J. van der Aa S. Andeweg A. C. Zaaraoui-Boutahar F. Goeijenbier M.. (2016). Influenza virus damages the alveolar barrier by disrupting epithelial cell tight junctions. Eur. Respir. J. 47, 954–966. 10.1183/13993003.01282-2015 26743480

[B30] Slepushkin V. A. Staber P. D. Wang G. McCray P. B. Jr. Davidson B. L. (2001). Infection of human airway epithelia with H1N1, H2N2, and H3N2 influenza A virus strains. Mol. Ther. 3, 395–402. 10.1006/mthe.2001.0277 11273782 PMC7106098

[B31] Spite M. Summers L. Porter T. F. Srivastava S. Bhatnagar A. Serhan C. N. (2009). Resolvin D1 controls inflammation initiated by glutathione-lipid conjugates formed during oxidative stress. Br. J. Pharmacol. 158, 1062–1073. 10.1111/j.1476-5381.2009.00234.x 19422383 PMC2785528

[B32] Sun Z. Wang F. Yang Y. Wang J. Sun S. Xia H.. (2019). Resolvin D1 attenuates ventilator-induced lung injury by reducing HMGB1 release in a HO-1-dependent pathway. Int. Immunopharmacol. 75, 105825. 10.1016/j.intimp.2019.105825 31437789

[B33] Sussan T. E. Gajghate S. Chatterjee S. Mandke P. McCormick S. Sudini K.. (2015). Nrf2 reduces allergic asthma in mice through enhanced airway epithelial cytoprotective function. Am. J. Physiol. Lung Cell Mol. Physiol. 309, L27–L36. 10.1152/ajplung.00398.2014 25957295 PMC4491510

[B34] To E. E. Erlich J. R. Liong F. Luong R. Liong S. Esaq F.. (2020). Mitochondrial Reactive Oxygen Species Contribute to Pathological Inflammation During Influenza A Virus Infection in Mice. Antioxid Redox Signal 32, 929–942. 10.1089/ars.2019.7727 31190565 PMC7104903

[B35] Uiprasertkul M. Kitphati R. Puthavathana P. Kriwong R. Kongchanagul A. Ungchusak K.. (2007). Apoptosis and pathogenesis of avian influenza A (H5N1) virus in humans. Emerg Infect. Dis. 13, 708–712. 10.3201/eid1305.060572 17553248 PMC2738443

[B36] Wang B. Gong X. Wan J. Y. Zhang L. Zhang Z. Li H. Z.. (2011). Resolvin D1 protects mice from LPS-induced acute lung injury. Pulm Pharmacol. Ther. 24, 434–441. 10.1016/j.pupt.2011.04.001 21501693

[B37] Wang H. Anthony D. Yatmaz S. Wijburg O. Satzke C. Levy B.. (2017). Aspirin-triggered resolvin D1 reduces pneumococcal lung infection and inflammation in a viral and bacterial coinfection pneumonia model. Clin. Sci. (Lond.) 131, 2347–2362. 10.1042/cs20171006 28779028

[B38] Wang M. Tan G. Eljaszewicz A. Meng Y. Wawrzyniak P. Acharya S.. (2019). Laundry detergents and detergent residue after rinsing directly disrupt tight junction barrier integrity in human bronchial epithelial cells. J. Allergy Clin. Immunol. 143, 1892–1903. 10.1016/j.jaci.2018.11.016 30500342

[B39] Wu H. M. Jiang Z. F. Ding P. S. Shao L. J. Liu R. Y. (2015). Hypoxia-induced autophagy mediates cisplatin resistance in lung cancer cells. Sci. Rep. 23, 5:12291. 10.1038/srep12291 PMC4511870 26201611

[B40] Yamaya M. Nishimura H. Hatachi Y. Yoshida M. Fujiwara H. Asada M.. (2011). Procaterol inhibits rhinovirus infection in primary cultures of human tracheal epithelial cells. Eur. J. Pharmacol. 650, 431–444. 10.1016/j.ejphar.2010.09.056 20940011

[B41] Zhang X. Wang T. Gui P. Yao C. Sun W. Wang L.. (2013). Resolvin D1 reverts lipopolysaccharide-induced TJ proteins disruption and the increase of cellular permeability by regulating IκBα signaling in human vascular endothelial cells. Oxid. Med. Cell Longev. 2013, 185715. 10.1155/2013/185715 24381712 PMC3870867

[B42] Zhao Q. Wu J. Lin Z. Hua Q. Zhang W. Ye L.. (2016). Resolvin D1 Alleviates the Lung Ischemia Reperfusion Injury via Complement, Immunoglobulin, TLR4, and Inflammatory Factors in Rats. Inflammation 39, 1319–1333. 10.1007/s10753-016-0364-9 27145782 PMC4951504

