# Supplementary figures and images for: ResolvinD1 Protects the Airway Barrier Against Injury Induced by Influenza A Virus Through the Nrf2 Pathway
Source: Front Cell Infect Microbiol. 2021 Feb 12;10:616475. doi: 10.3389/fcimb.2020.616475 (PMC7907644; doi:10.3389/fcimb.2020.616475)

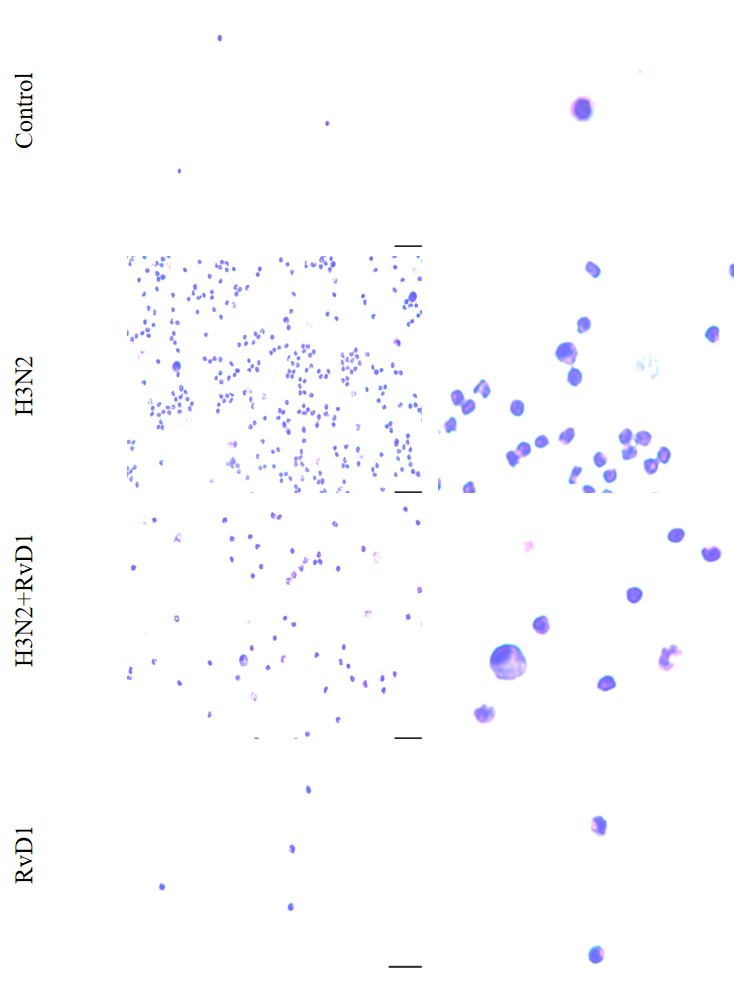

Supplement: Supplementary file 1 [file Image_1.jpeg]
